# Supplementary material for: An Integrated In Silico and In Vitro Approach for the Identification of Natural Products Active against SARS-CoV-2
Source: Biomolecules. 2023 Dec 28;14(1):43. doi: 10.3390/biom14010043 (PMC10813393; doi:10.3390/biom14010043)
Supplement: Supplementary file 1 [file biomolecules-14-00043-s001.zip › biomolecules-2774830-supplementary.pdf]

biomolecules

Supporting Information

An Integrated In Silico and In Vitro Approach for the Identification of Natural Products Active against SARS-CoV-2

Rosamaria Pennisi [1,*], Davide Gentile [2,*], Antonio Rescifina [3], Edoardo Napoli [4], Paola Trischitta [1,5], Anna Piperno [1] and Maria Teresa Sciortino [1]

[1] Department of Chemical, Biological, Pharmaceutical and Environmental Science, University of Messina, Viale Ferdinando Stagno d'Alcontres 31, 98166 Messina, Italy; paola.trischitta@studenti.unime.it (P.T.); anna.piperno@unime.it (A.P.); mtsciortino@unime.it (M.T.S.)
[2] Department of Chemistry, Materials and Chemical Engineering "G. Natta", Politecnico di Milano, Via Mancinelli 7, 20131 Milano, Italy
[3] Department of Drug and Health Sciences, University of Catania, V.le A. Doria 6, 95125 Catania, Italy; arescifina@unict.it
[4] Istituto di Chimica Biomolecolare—Consiglio Nazionale delle Ricerche, 95126 Catania, Italy; edoardo.napoli@icb.cnr.it
[5] Department of Chemistry, Biology and Biotechnology, University of Perugia, Via Elce di Sotto 8, 06123 Perugia, Italy
* Correspondence: rpennisi@unime.it (R.P.); davide.gentile@polimi.it (D.G.)

Samples preparation

Commercially available cynarin (CY), rosmarinic acid (RA), folic acid (FA), and luteolin-7-O-glucuronide (L7OG) were used to prepare the respective stock solutions. Specifically, L7OG was dissolved in DMSO to obtain a 5.5 mM solution, RA was dissolved in water to obtain an 8.0 mM solution, CY was dissolved in methanol to obtain a 4.7 mM solution, and FA was dissolved in ethanol to obtain an 8.7 mM solution.

Viability assay of natural compounds

VERO cells were exposed to different concentrations of L7OG, CY, FA, and RA for 72 h and incubated with CCK8 tetrazolium salt for 4 h before measuring the absorbance values in a microplate reader. The percentage of cellular viability was calculated and compared to untreated cells. The 50% cytotoxic concentration (CC_{50}) was reported in Table S1.

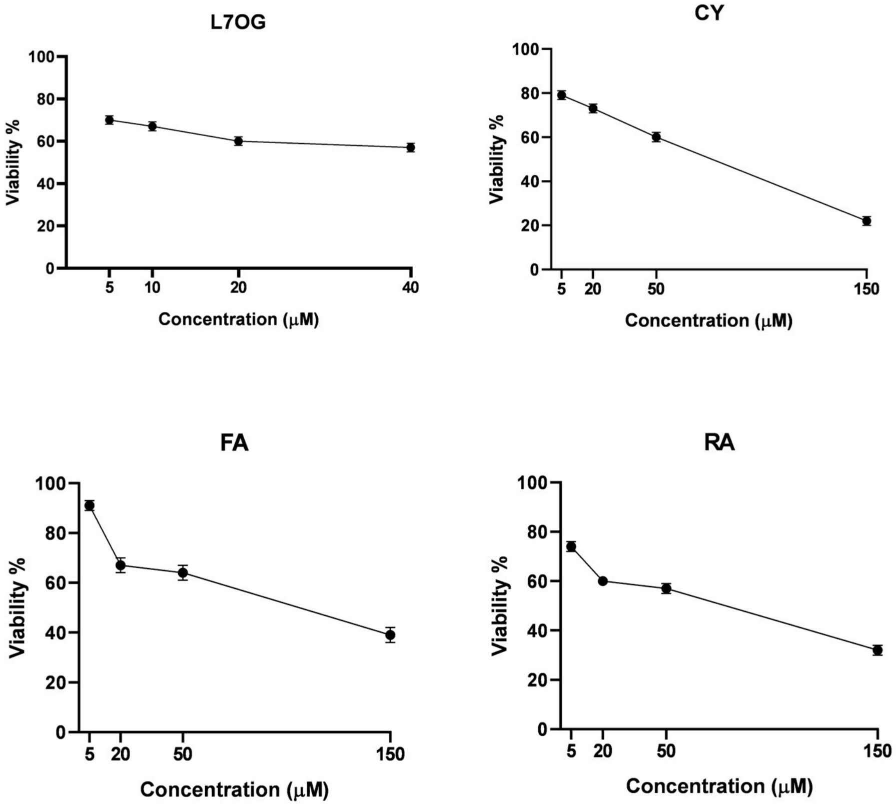

Figure S1. Cellular viability assay following treatment with different L7OG, CY, FA, and RA concentrations. VERO cells were incubated for 24 h with various concentrations of natural compounds, as reported in the material and methods section. Results represent the mean of three biologically independent experiments ± SD.

Table S1. Cytotoxicity (CC_{50}) values (μM) of natural compounds on VERO cells.

Compound	CC_{50}
L7OG	55.71
CY	91.47
FA	107.16
RA	78.30

Table S2. Structures and calculated free binding energies (ΔG_B, in kcal/mol) of the selected compounds.

Compound	$3CL^{pro}$ PDB ID: 6LU7	PL^{pro} PDB ID: 6W9C	α-RBD PDB ID: 7Y42	o-RBD PDB ID: 7WK2	o-RBD/ACE2 complex PDB ID: 7WBL
L7OG	−7.1	−7.7	−8.5	−6.0	−8.6
CY	−6.6	−6.5	−6.5	−5.1	−6.6
FA	−7.0	−7.8	−8.8	−6.1	−8.9
RA	−6.7	−6.1	−6.4	−5.1	−6.7

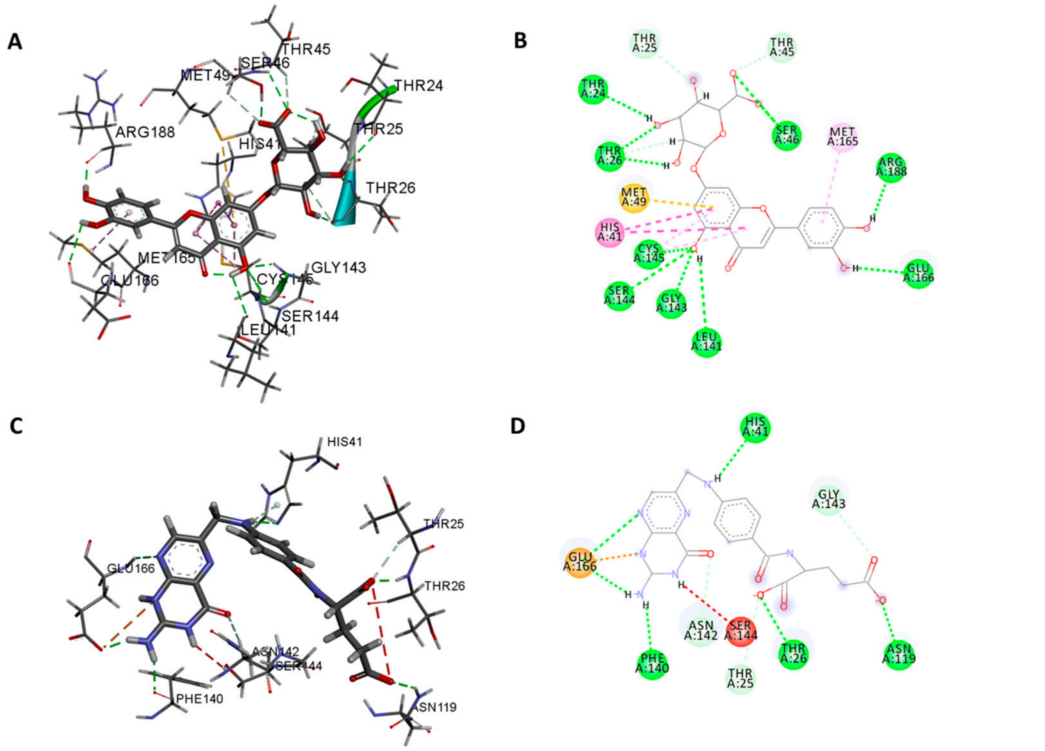

Figure S2. (**A**) Interaction profile of the best-docked poses for L7OG/$3CL^{pro}$ (**B**) and 2D diagram interaction profile. (**C**) Interaction profile of the best-docked poses for FA/$3CL^{pro}$ (**D**) and 2D diagram interaction profile.

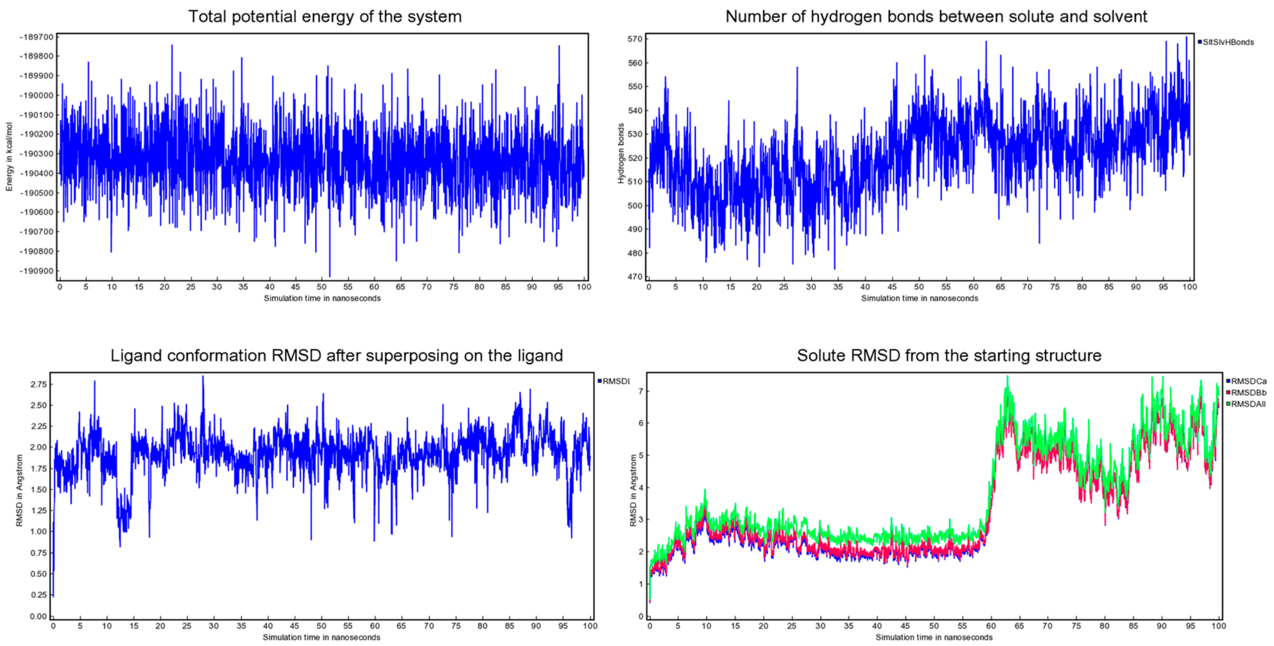

Figure S3. L7OG/3CL^{pro}. Total energy (top left) and the number of hydrogen bonds between solute and solvent of 3CL^{pro} enzyme and its complexes with ligand (top right). RMSD of the ligand (bottom left) and solute RMSD from the starting structure inside the binding pocket of 3CL^{pro} enzyme (bottom right).

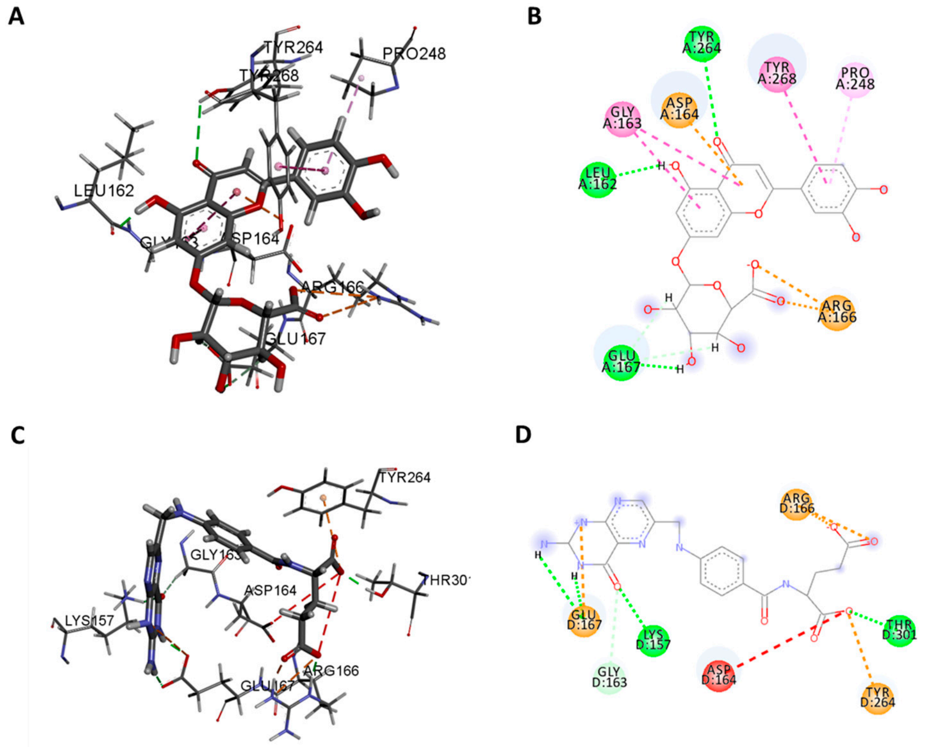

Figure S4. (**A**) Interaction profile of the best-docked poses for L7OG/PL^{pro} (**B**) and 2D diagram interaction profile. (**C**) Interaction profile of the best-docked poses for FA/PL^{pro} (**D**) and 2D diagram interaction profile.

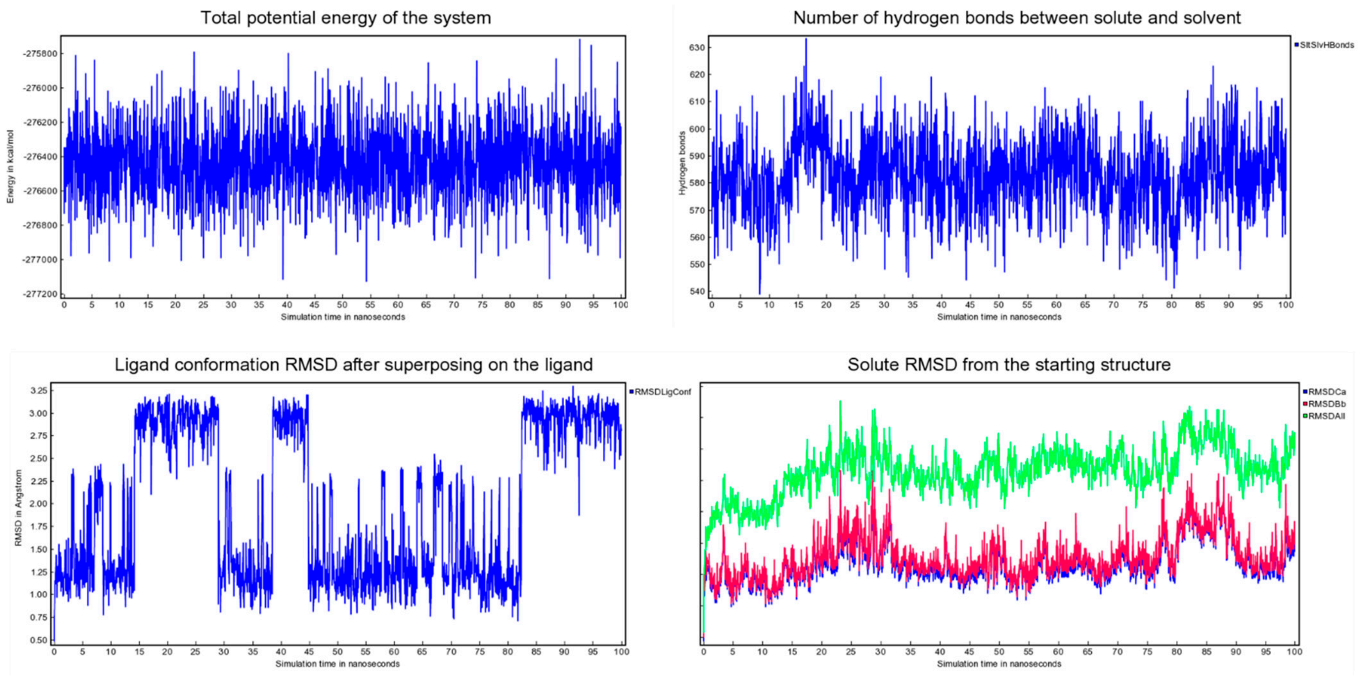

Figure S5. L7OG /PL^{pro}. Total energy (up-left) and the number of hydrogen bonds between solute and solvent of PL^{pro} enzyme and its complexes with ligand (top right). RMSD of the ligand (bottom left) and solute RMSD from the starting structure inside the binding pocket of PL^{pro} enzyme (bottom right).

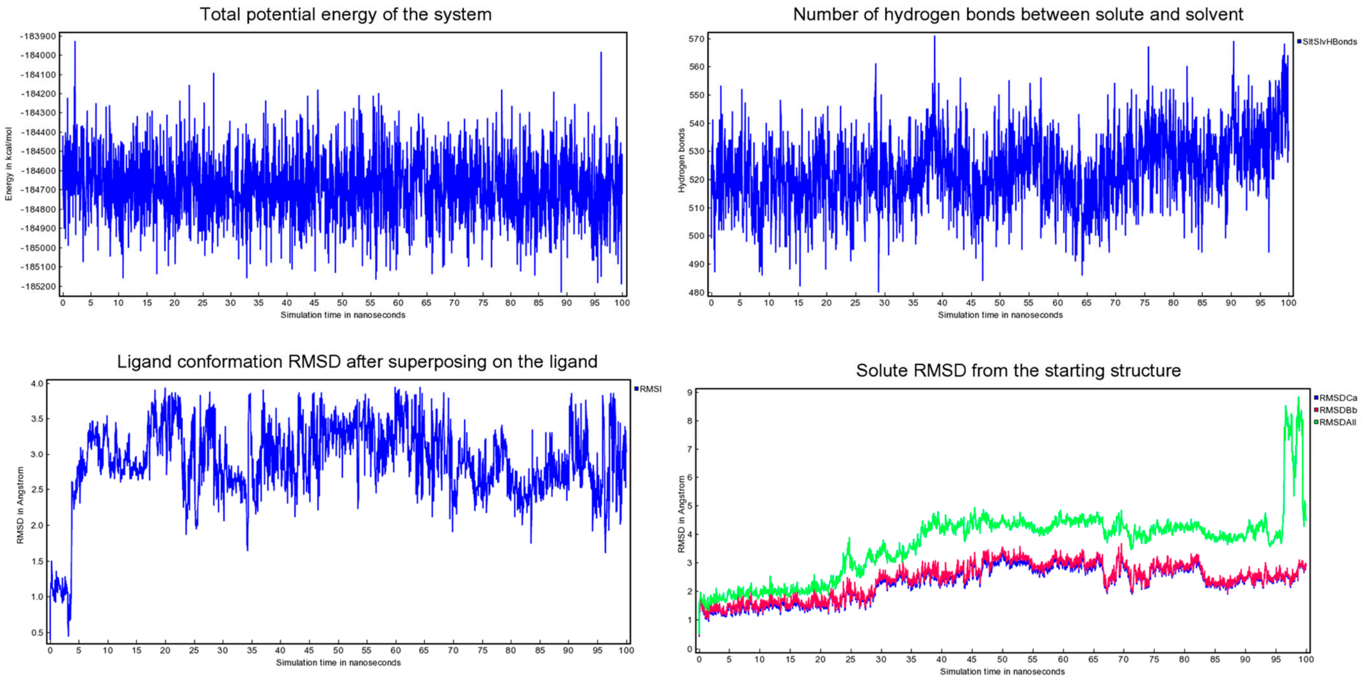

Figure S6. FA/PL^{pro}. Total energy (up-left) and the number of hydrogen bonds between solute and solvent of PL^{pro} enzyme and its complexes with ligand (top right). RMSD of the ligand (bottom left) and solute RMSD from the starting structure inside the binding pocket of PL^{pro} enzyme (bottom right).

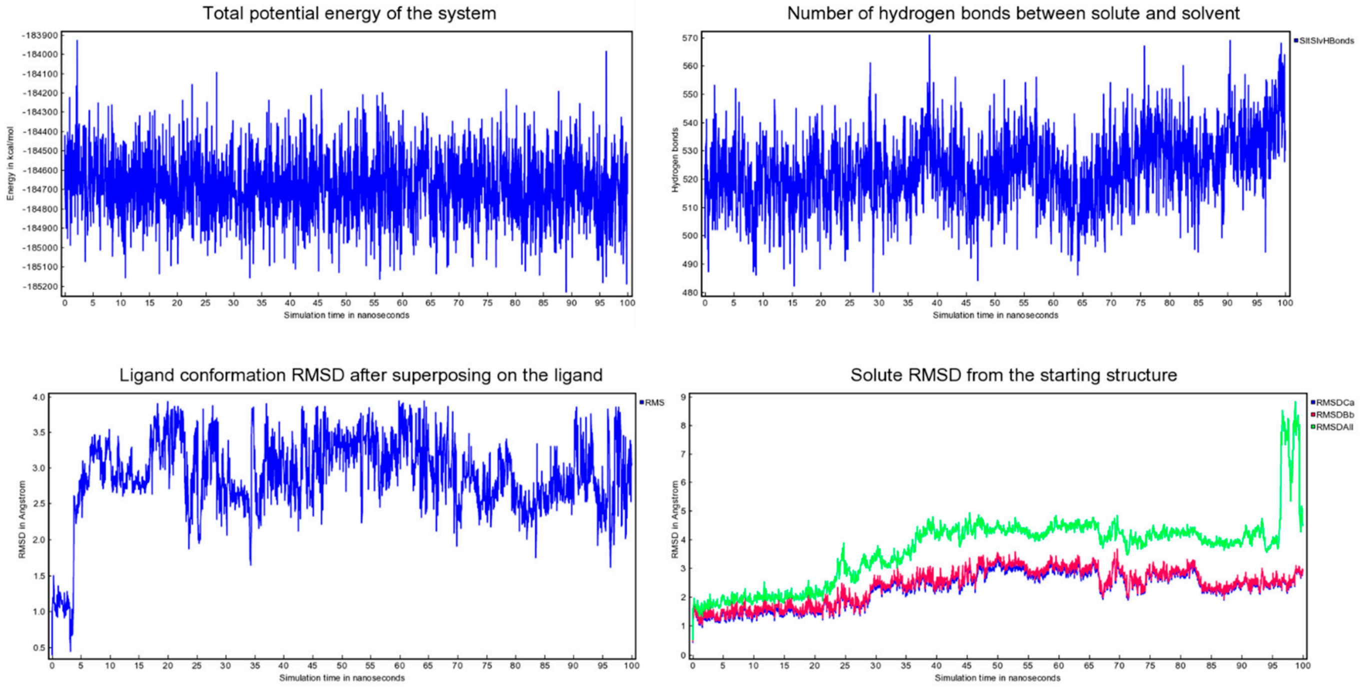

Figure S7. FA/3CL^{pro}. Total energy (up-left) and the number of hydrogen bonds between solute and solvent of 3CL^{pro} enzyme and its complexes with ligand (top right). RMSD of the ligand (bottom left) and solute RMSD from the starting structure inside the binding pocket of 3CL^{pro} enzyme (bottom right).

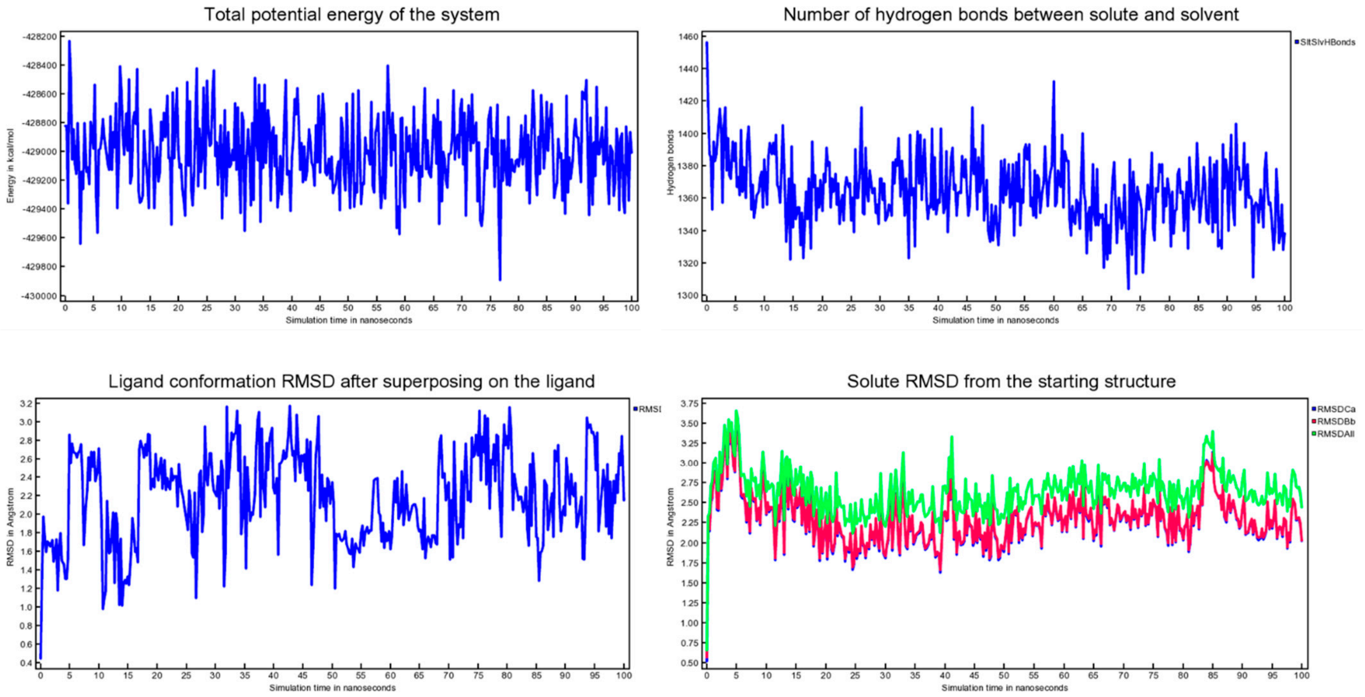

Figure S8. FA/o-RBD/ACE2 complex. Total energy (up-left) and the number of hydrogen bonds between solute and solvent of o-RBD/ACE2 complexes with ligand (top right). RMSD of the ligand (bottom left) and solute RMSD from the starting structure inside the binding pocket of o-RBD/ACE2 complexes (bottom right).
